# Review about Non-Lipid Components and Minor Fat-Soluble Bioactive Compounds of Almond Kernel

**DOI:** 10.3390/foods9111646

**Published:** 2020-11-11

**Authors:** José M. Roncero, Manuel Álvarez-Ortí, Arturo Pardo-Giménez, Adrián Rabadán, José E. Pardo

**Affiliations:** 1 Higher Technical School of Agricultural and Forestry Engineering, University of Castilla-La Mancha, Campus Universitario, s/n, 02071 Albacete, Spain; manuel.alvarez@uclm.es (M.Á.-O.); adrian.rabadan@uclm.es (A.R.); jose.pgonzalez@uclm.es (J.E.P.); 2 Mushroom Research, Experimentation and Service Centre, C/Peñicas, s/n, Apartado 63, Quintanar del Rey, 16220 Cuenca, Spain; apardo.cies@dipucuenca.es

**Keywords:** tree nuts, chemical composition, proteins, carbohydrates, minerals, phytochemicals, polyphenols, antioxidants, volatile compounds

## Abstract

This work presents a bibliographic review about almond kernel non-lipid components, in particular about the protein fraction, the carbohydrates and the mineral fraction. In addition, other fat-soluble phytochemicals which are present in minor concentrations but show important antioxidant activities are reviewed. Almond kernel is a rich protein food (8.4–35.1%), in which the globulin–albumin fraction dominates, followed by glutelins and prolamins. Within the almond kernel protein profile, amandine dominates. Free amino acids represent a small amount of the total nitrogen quantity, highlighting the presence of glutamic acid and aspartic acid, followed by arginine. Carbohydrates that appear in almond kernels (14–28%) are soluble sugars (mainly sucrose), starch and other polysaccharides such as cellulose and non-digestible hemicelluloses. Regarding the mineral elements, potassium is the most common, followed by phosphorus; both macronutrients represent more than 70% of the total mineral fraction, without taking into account nitrogen. Microminerals include sodium, iron, copper, manganese and zinc. Within the phytochemical compounds, tocopherols, squalene, phytosterols, stanols, sphingolipids, phospholipids, chlorophylls, carotenoids, phenols and volatile compounds can be found.

## 1. Introduction

The almond is the most cultivated nut in the world, where the estimated annual production exceeds 3 million tons [1]. Most of the world’s production is concentrated in three regions, which include California, the Mediterranean Basin and the Middle East, although almond cultivation is also increasing in the Southern Hemisphere, in countries such as Australia or Chile.

Almond tree, *Prunus dulcis*, belongs, taxonomically, to the *Amygdalus* subgenus inside the *Prunus* genus, the *Rosaceae* family and the order *Rosales* [2]. Its cultivars are classified depending on the hardness of the shell. Soft and medium-hard shell cultivars, like Non Pareil and Guara, respectively, show low resistance to attacks by pests and are more susceptible to rancid oxidation, but show high kernel yields (55% and 35–40%, respectively) [3]. On the other hand, hard shell varieties present the lowest kernel yield (<25%), but they maintain in a better way the organoleptic and commercial characteristics, highlighting the importance of Marcona and Desmayo Largueta cultivars. Physical parameters are useful for cultivar determination even when the nuts are grown in the same conditions.

From the botanic point of view, the almond tree nut is a drupe. It is formed by the evolution of the ovary walls, which develop into the pericarp (hull), an outer layer that is formed by a pulpy and very fibrous tissue, that can be divided into the exocarp (thin and pubescent) and the mesocarp (thickest); and a lignified interior layer that creates a heavy to less heavy coat, the endocarp (shell). At maturity, the pulpy mesocarp dries and opens by its ventral suture, releasing the lignified endocarp. The seed, which constitutes the edible kernel and the commercial part of the nut, occupies the inner part, surrounded by the endocarp. The kernel contains the embryo coated by the teguments [2].

Almond consumption has been found to be associated with many health benefits [4], especially related to the reduction of the cardiovascular diseases risk, but also with effects on other pathologies, such as hypertension, diabetes mellitus or metabolic syndrome. These activities are generally attributed to the lipid fraction, where the fatty acid profile has a predominant role, but also minor compounds such as polyphenols and phytosterols may be involved. Moreover, recent studies have explored the effect of other nutritional compounds like fiber on gut microbiota [5] or the antioxidant capacity of the protein fraction [6].

Regarding the chemical composition of almond kernels, the fatty acid profile has been extensively studied and characterized. However, the information about other minor compounds and the non-lipid fraction, in which a large quantity of nutrients are found, is more scarce, and is generally presented separately, so it is difficult to find those gathered to obtain an overall view of the content of all these compounds in the almond kernel. Therefore, this review aims to present data on the composition of the non-lipid fraction as well as other less studied minor compounds in almond kernels, to provide an overview of all these compounds with potential benefits on human health.

## 2. Chemical Composition of Almond Kernel

**Figure 1:**
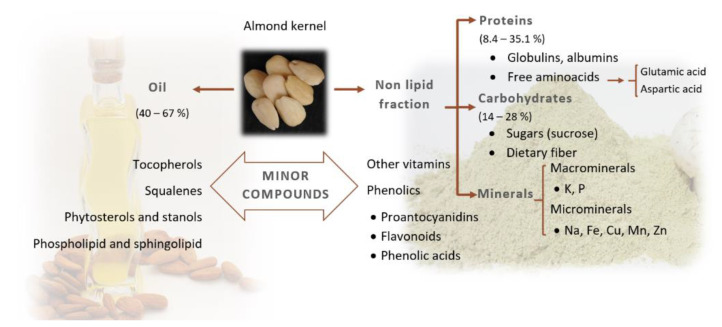
Chemical composition of almond kernel.

The main fractions that can be found in almond kernels, other than water, are the lipid fraction, the protein fraction, carbohydrates and the mineral fraction. A numerous group of compounds called phytochemicals should also be added, because even though they appear in low quantities, they have a main role in almond quality. The proportion of these compounds changes according to the cultivars, the cultivation system and the geographical origin [7,8,9,10,11,12] (Figure 1).

Precise knowledge about almond kernel composition is of great interest from the commercial, industrial and nutritional points of view, especially taking into account the variability that exists between different cultivars. In Yada et al. [8], total lipid values between 40 and 67 g/100 g of dry almond weight and between 35 and 66 g/100 g of almond fresh weight (f.w.) were reported. Almond oil is mainly composed of mono- and diunsaturated fatty acids [13]. In the case of total proteins (considering a conversion N factor of 5.18), the values oscillated between 14 and 61 g/100 g of almond fresh weight, and in the case of soluble sugars, values between 1.8 and 7.6 g/100 g of dry almond and between 2.5 and 12 g/100 g of fresh almond have been reported.

Regarding phenotypic correlations, a negative correlation was found between the oil and the total protein content [14]. This interdependence can be explained biochemically, because both fractions are formed during the ripening process from carbohydrates, which are abundant in the early stages of seed development but decrease over the ripening process [15].

In the existing literature, a clear evolution of the topic treatment can be observed. The first works about the chemical composition of almond kernels started appearing in the 1950s [16,17], providing data about the main fractions, without discrimination between cultivars and origins. In the decades of the 1970s and 1980s, works about the chemical composition appeared, referring to defined cultivars and providing information about fatty acids, amino acids, mineral salts and soluble sugars compositions [15,18,19]. Works studying the influence and effect of different labor systems, the place of origin and the harvest year on almond chemical composition also appeared.

From 1990, a step forward can be observed, related to the use of advanced statistical treatments, in such a way that not only composition data are given, but it is also tried to bring together genotypes that have a similar response and present close values to commercial effects [7,20,21,22]. In addition, food origin determination appears as a main target in food quality control and safety [23].

In the new century, the approach to the chemical composition of almond kernels, which can be applied to the rest of nuts, is focused on minority components, called phytochemicals. It has been shown that almond kernels present a wide range of substances with high nutritional value or with effects on health on one side [24,25,26] and with antioxidant effects on the other [27,28,29]. The interest aroused by these substances has boosted the development of new methods for their determination, increasing exponentially the published articles about them. Another important source of data is due to the recent interest in almond oil extraction as virgin edible oil [11,12,13,30,31,32,33,34,35]. In this sense, almond oil has been widely characterized, but oil extraction industries generate a by-product derived from the grinding of the pressing cake, which originates a partially defatted flour where the non-lipidic fraction takes on a special relevance. These flours have been reported to have promising uses in the culinary industry to enhance the nutritional properties of various products [36,37], or in mushroom cultivation, where it can be added as a nutritional supplement [34].

## 3. Protein Fraction of Almond Kernel

Almond kernel is a protein-rich food (second fraction in importance after the lipid fraction), but its content presents differences depending on the cultivar, weather conditions and cultivation area [9,14,16,32,34,38,39,40,41,42].

Table 1 shows the protein content of almond kernel samples with different origins found in relevant published articles. The percentage of variation ranges from 8.4%, found in Spanish samples [14], to 35.1%, found in Moroccan samples [42]. The differences in the protein content found in different samples may be related to the methods used in the analysis. To calculate the protein content, typically a specific conversion factor of nitrogen to protein of 5.18 is used [43], since amandine, which is the dominant protein in almond, is a globulin that contains 19.3% of nitrogen [44]. However, other studies use the general conversion factor (6.25), based on the nitrogen content of most common proteins, which could lead to overestimate the protein content. This point could explain some discrepancies found within the data. For this reason, data regarding total nitrogen would be more useful to compare samples from different origins.

Font i Forcada et al. [61] found that two quantitative trait loci (QTL) controlled the total protein content. The first marker LG6, located in the lowest part of the almond linkage groups, had a logarithm of the odds (LOD) values of 3.21 and explained a phenotypic variance of 17%. The second QTL was found in the lowest part of LG7 and had a similar effect, with an LOD of 3.18 explaining a phenotypic variance of 16.6%.

Nitrogen total content of almond samples has shown different percentages: 3% [15], 4.06% [62], 4.23% [54] and 4.62% [63].

### 3.1. Protein Profile

Proteins are classified depending on their solubility in albumins (soluble in water and dilute solutions), globulins (classified into euglobulins—soluble in dilute solutions, acids and alkalis and insoluble in water, and pseudoglobulins—moderately soluble in these solutions), prolamins (soluble in solutions with 50–90% ethanol), glutelins (soluble in dilute acids and alkalis) and scleroproteins (insoluble in all mentioned solvents).

Saura et al. [15] found that the protein dominant fraction was that composed by globulins–albumins, with about 90% in all studied samples. On the other hand, glutelins accounted for between 4 and 11% of total proteins, while prolamins were found below 0.4% in all cases.

Within the protein profile, amandine is dominant, also known as the almond major protein (AMP), which represents 65% of total proteins of almond that can be extracted in aqueous medium [64]. This protein is the main component responsible for food allergies caused by almonds, due to the antigenic activity that it presents. It is an ideal marker to detect traces of almond in foods [24].

### 3.2. Free Amino Acids

Together, free amino acids (AAs) represent a small quantity of total nitrogen matter (1%), which matches with the low content of non-protein nitrogen; consequently, the total amino acids content in almond kernel is a good approximation of the total protein content. The main free amino acids found in almond kernel are glutamic acid and aspartic acid (including glutamine and asparagine), followed by arginine. Phenylalanine, alanine, serine and threonine are also present although with lower quantities [15,65]. Amrein et al. [66] also found that aspargine was the main free amino acid in raw almond kernel (20–50% of total free amino acids).

The free amino acids fraction has been used for the characterization of almond cultivars [65,67]. These free amino acids are important due to their contribution to food taste and for being precursors of aromatic components and colored substances that are produced during the obtention, preparation and storage of food.

Esteban [18] found a higher content in almost all AAs in cultivars grown in northwest Spain compared to those grown in the southwest, which was related to the lower content of fats in the northwest cultivars.

### 3.3. Essential Amino Acids

Humans are unable to synthesize eight AAs that need to be necessarily obtained through diet, and these are known as essential amino acids. Arginine and histidine must be included in this group with essential amino acids (threonine, methionine, valine, isoleucine, leucine, phenylalanine, tryptophan and lysine) because they are considered essential for children but not for adults. In addition, cysteine and tyrosine are considered semi-essential amino acids, due to the sparing effect they have on methionine and phenylalanine, respectively.

Esteban [18] and, later, Saura et al. [15] found that the most abundant essential amino acid in almond kernel was arginine, with an average value of 524 mg/g of N. Essential AAs represent 28% of total amino acids. Regarding the limiting amino acids in almond kernel, comparing to egg, they found that the first one would be lysine, followed by threonine. Ahrens et al. [45] found that methionine together with cysteine was the limiting amino acid, followed by lysine and threonine. More recently, House et al. [68] considered that Ahrens et al. had underestimated the total sulfur AA content due to a method issue. They found lysine as the limiting AA.

On the other side, the inhibitory activity of trypsin was evaluated, as well as the hemagglutinating activity, not being detected in analyzed samples.

The digestibility of the protein and ultimate utilization of the constituent AAs for metabolic functions are equally important in assessing protein quality [68]. Amino acid score (AAS) together with digestibility is a parameter that allows calculating the protein digestibility-corrected amino acid score (PDCAAS), which can be used to properly establish the protein quality index. In this sense, although almond kernel proteins show a high degree of digestibility, higher than 80% in all analyzed samples measured as protein digestibility in vivo, when the protein digestibility index is corrected by the AAS, it results in low quality [45]. On the other hand, House et al. [68] obtained better values. This kind of study has not been contemplated in the rest of the previously cited research.

## 4. Carbohydrates in Almond Kernel

The carbohydrates from almond kernel are soluble sugars, starch and other polysaccharides such as celluloses and hemicelluloses that are non-digestible, but they have physical effects in the intestinal tract with benefits for human health [8]. The total carbohydrates content ranged from 14% to 28% (Table 1). The sugars that can be found in almond kernel, although not found in high concentrations, are enough to provide the sweet flavor to almonds.

### 4.1. Sugars

Nuts are characterized by low quantities of soluble sugars, that range from 2.6% to 7.9% (Table 1). The soluble sugars fraction suffers important quantitative variations depending mainly on the cultivar, but also on the origin and even harvest time [9,18,69,70]. Most sugars are not reducing, with sucrose representing more than 90% of the total sugars. Raffinose, inositol, sorbitol, fructose and glucose were also detected [55,56,71]. Differences found between commercial and regional cultivars about sugars composition, especially sucrose, may be used to establish a sugar profile as an indicator of almond kernel quality.

Moreover, Nanos et al. [69] found galactose (reducing sugars), melezitose (tri-saccharide) and stachyose (tetrasaccharide). Late-harvested almonds had lower total amounts of these sugars than early-harvested ones.

The fact that the main sugar is sucrose is due to its preferential production and its accumulation in almond kernel during ripening and to the fact that many minor sugars constitute a substrate for the synthesis of sucrose [57]. Kazantzis et al. [55] observed that almond kernels early-harvested (green mesocarp surface at 90%) had a lower content in sucrose and higher content in inositol than more ripened almonds (brown mesocarp surface at 90%). Trees subjected to irrigation and compost treatments also produce almond kernels with the highest content in sucrose and glucose. This could reduce their water content, causing a higher concentration of compounds such as sugars [57]. In contrast, a recent study by Lipan et al. [72] showed that almost all morphological and physical-chemical parameters were unaffected by water stress.

Regarding the total sugars, a value of 4.63 g/100 g of peeled almonds was found, distributed as 4.46 g of sucrose, 0.03 g of glucose and 0.14 g of maltose [43]. A review of a worldwide collection of almond samples found a soluble sugars content ranging from 1.80 to 7.60 g/100 g f.w. [8]. On the other side, when data are referred to almonds with skin, total values fall to 4.35 g/100 g, and are distributed as follows: 3.95 g of sucrose, 0.17 g of glucose and 0.04 g of maltose, appearing with 0.11 g of fructose and 0.07 g of galactose.

### 4.2. Starch

Although starch is the main reserve carbohydrate in many fruits and seeds, in almond kernel, it does not reach remarkable values. Thus, this compound has not attracted much attention among researchers. Ruggeri et al. [59] found a percentage of 0.4% to 1.4%. The Agriculture Department of the U.S.A. indicated a starch value of only 1.0 g/100 g of skin peeled almond kernels [43].

### 4.3. Fiber

Fiber is a heterogeneous mix of polysaccharides (cellulose, hemicelluloses, gums and mucilages and pectin substances) and non-polysaccharides (lignin, non-digestible proteins and other). Terms commonly used to define it are as follows: crude fiber, composed of cellulose (50–80%), hemicellulose (~20%) and lignin (10–50%); neutral detergent fiber (NDF), consisting of cellulose, hemicellulose and lignin; acid detergent fiber (ADF), consisting of cellulose and lignin; and acid detergent lignin (ADL) [73]. On the other side, dietary fiber can be defined as a group of components that are not digested by enzymes in the human gastro-intestinal tract, and as being mainly composed of cellulose, hemicellulose, lignin, pectin and non-digestible proteins.

The content in neutral detergent fiber, whose fundamental components are cellulose, lignin and hemicellulose, provides the value of dietary fiber, while acid detergent fiber, whose fundamental components are cellulose and lignin, provides the crude fiber value.

Barreira et al. [46] reported data of neutral detergent fiber between 2.9% and 3.2%, depending on the selected cultivar. However, dietary fiber content in almonds ranged from 3.3% to 16% (Table 1). Kodad [22] calculated a variation coefficient of 9.81% for dietary fiber. Significant differences between selected cultivars, years and the interaction “genotype” × “year” have been found regarding dietary fiber content, which confirms the large variability of this character between genotypes and years. Yada et al. [9] found that the effect of harvest year on dietary fiber was highly significant (*P* < 0.01), to the point of having blocked the observation of cultivar differences. In addition, some other agronomic treatments may have an effect on the content of dietary fiber. For example, when organic fertilizers were applied, higher fiber content was observed in the fruit than when an inorganic fertilizer was employed [57].

Crude fiber concentration in almond kernel also shows high variability. First references indicated low crude fiber contents, about 2% or 3% of dry matter [15,16], while other recent results reached contents of 5.81% [22], probably due to improvements in analytical techniques.

## 5. Mineral Fraction of Almond Kernel

### 5.1. Ashes

Mineral content is sometimes expressed as the ash content, which is the inorganic residue that remains after the incineration of the plant tissues. Almond kernels contain approximately 3 g ash/100 g of fresh weight [74,75]. These values may vary depending on the study considered (Table 2), between 2.3% [9] and 5.0% [51].

The sum of mineral elements is sensibly lower than the ash content, with percentages around 60%, which is fundamentally explained because the oxygen associated with these minerals is not counted in the ashes obtained by calcination [15]. According to Esteban [18], the percentge of all minerals, excluding nitrogen, represents between 51.3% and 55.2% of total ash content.

### 5.2. Macrominerals

Macrominerals refer to those minerals that are needed in quantities higher than 100 mg/day. On the other hand, those that are needed in small quantities are called microminerals, oligo elements or trace elements. Table 2 shows the average value of the main mineral elements (macrominerals and microminerals) found in almond kernel.

Potassium is the major element in all studies, except the one carried out by Prats [7], followed by phosphorus. Both elements represent 70% of the mineral fraction, not counting nitrogen. The next in importance are calcium and magnesium with very close values, in such a way that in some samples, one is higher and in others the opposite happens [15,18]. Globally, the mean magnesium values are higher than calcium values, and both represent half the phosphorus content, or even less [15].

Sulfur also appears in high amounts, although it is an element that is not commonly analyzed in comparison with the previous ones. Its values vary greatly depending on the study, probably due to the different methods applied for its determination. Prats [7] found higher values, comparable to phosphorus values. Macronutrients aggregation, not counting nitrogen, represents large percentages which are almost identical between cultivars, ranging from 98.0% to 98.7% of total minerals. Among Chinese wild almond species, potassium contents between 534 and 663 mg/100 g, calcium contents between 80 and 229 mg/100 g and magnesium contents between 194 and 239 mg/100 g have been found [77].

### 5.3. Microminerals or Trace Elements

Main microminerals or trace elements found in almond kernel are sodium, chlorine, iron, copper, manganese and zinc (Table 2). Sodium and chlorine are those that appear in higher proportion [15,16,54], followed by iron and zinc contents, which also show important values. In this case, as it happened with calcium and magnesium, for some authors, the content of iron is higher, and for others, the zinc content, but generally the quantity, is lower than 5.5 mg/100 g. Nevertheless, attention should be paid to the high contents in iron and zinc found by Ozcan et al. [40] and Aslantas et al. [54], respectively. Among Chinese wild almond species, iron contents between 4.6 and 6.0 mg/100 g and zinc contents between 4.1 and 5.6 mg/100 g have been found [77].

Other elements found in almond kernel, although in minor concentrations, include molybdenum that ranges from 4 to 30 µg/100 g, boron which ranges between 0.18 and 2.9 mg/100 g [15,16,78], chromium ranging between 0.04 [79] and 0.17 mg/100 g [78], aluminum ranging between 0.83 [79] and 2.2 mg/100 g [78], nickel with 0.034 mg/100 g [79] and selenium with 0.004 mg/100 g [51].

Some references to toxic heavy metals have also been found [50,51,79]. Even though some heavy metals such as cobalt, copper, chromium, manganese and nickel are needed for humans in small proportions, others may be carcinogenic or toxic, affecting the central nervous system (manganese, mercury, lead, arsenic), kidney or liver (mercury, lead, cadmium, copper), or the skin, bones or teeth (nickel, cadmium, copper, chromium).

## 6. Phytochemical Compounds of Almond Kernel

Phytochemicals, also known as bioactive compounds, are mainly additional nutritional compounds that can be found in certain foods, and that show an important and interesting physiological activity with positive effects on human health, which makes them very valuable elements for the scientific community and the food industry.

Several thousands of phytochemicals have been reported, some of them having a strong antioxidant activity (catechin, quercetin, tannin, ellagic acid, chlorogenic acid, cyanidin, etc.) [80], which are added to the already known antioxidant nutrients (vitamins A, C, E, selenium, etc.).

Phytochemicals comprise the following chemical groups: carotenoids, phenolic compounds, organosulfur compounds, some nitrogen compounds and alkaloids. Bolling et al. [81] added a carbohydrates group to this classification, the phytates, and together with the carotenoids, they include other unsaponifiable compounds of the lipid fraction.

### 6.1. Tocopherols (Vitamin E)

Tocopherols, or vitamin E, are a group of soluble compounds that includes four tocopherols (designated as α, β, γ and δ) and four tocotrienols (designated as α, β, γ, y and δ) [80]. Tocopherols are natural mono-phenolic components with different antioxidant activity, which have several homologues depending on the position and number of methyl radicals. Their main biochemical function is probably the protection of polyunsaturated fatty acids against peroxidation. A good number of scientific studies focused, in the first instance, on the tocopherol content and its effect on the maintenance of oil properties. Almond kernel is considered one of the richest foods in α-tocopherol [82,83].

Tocopherol content in almonds shows a wide range of variability, as summarized in Table 3. The form with higher concentration in almond kernel oil is α-tocopherol. Variability depends on almond genotypes (cultivars), climatic conditions and environmental conditions. Kodad et al. [10], in a study about 44 Spanish cultivars, for two consecutive years, found a large variability in tocopherol concentrations, in almond oil, with a significant effect of both the genotype, the year and the interaction genotype × year. The main source of variability appeared due to the genotype. The geographical origin was significant with higher concentrations of tocopherols in almond populations with a mountainous origin, probably due to the empiric selection to increase the shelf life, since tocopherol retards the rancidity appearance. Abiotic stress leads to higher tocopherol contents due to its protective role. Similar conclusions reached Zhu [28], after analyzing samples of cultivars from Australia, Spain and the United States, and Yada et al. [9], with Californian cultivars from different regions. Besides, as the trees matured from one year to the next, the vitamin E concentration increased [10]. The obtained results also show that the homologues α and δ are those that present higher variability. Higher concentrations found by Maestri et al. [30] in the Argentine Northeast, where the kernel development matches mainly with spring and summer, with medium temperatures that are warmer to those typically observed in the Mediterranean region, can explain these values.

### 6.2. Vitamins

Most studies about vitamin content in almond have been focused on vitamins with an antioxidant effect, particularly vitamin E. However, almond kernels are a good source of vitamins B1 (thiamine), B2 (riboflavin), B6 (pyridoxine) and niacin (Table 3). Some kernel processing operations, like roasting or blanching (to a lesser extent), may result in vitamin loss due to the temperature effect on vitamin degradation [89].

### 6.3. Squalenes

Squalenes are polyunsaturated acyclic hydrocarbons with a triterpenoid lipophilic structure, similar to the vitamin E structure, and they contribute to the oxidation stability of vegetable oils because they prevent peroxidation of fats acting mainly against peroxyl radicals.

Squalene acts as a biosynthetic precursor to all steroids in plants and animals. However, Cherif et al. [90] detected a dramatic decrease in sterols at the 10–12th maturation week that suggested there was the absence of the synthesis of novo sterols from squalene which was maintained an enzymatic activity until the end of maturity. Squalene has important beneficial effects on health, such as decreasing the risk for various cancers and reducing serum cholesterol levels [91]. Squalene contents in almond oils ranged from 37.9 to 114.2 µg/g of oil (Table 4).

### 6.4. Phytosterols and Stanols

Phytosterols or plant sterols have a structure similar to cholesterol, while stanols are saturated sterols. They can be found in almond kernel, in free form or esterified with fatty acids [80]. β-sitosterol lowers cholesterol levels, enhances immunity and has anti-inflammatory, antipyretic and anti-carcinogenic effects (prostate essentially) [92].

Sterols are the most abundant class of compounds in the unsaponifiable matter. Desmethylsterols are the most commonly analyzed group, being *β*-sitosterol the main desmethylsterol with values of 95.5 % of total phytosterols [12], although with significant differences among genotypes [70,90,94]. As regards methylsterols, citrostadienol is the main compound, and regarding dimethylsterols, the total amount was around 30 µg/g [94].

β-sitosterol and campesterol are the dominant sterols in almond kernel. Δ5-Avenasterol, Δ7-Stigamasterol and stigmasterol are well represented in almond oils. Campestanol is the main stanol (Table 4). β-sitosterol is fundamentally found in almond kernel skin, while stigmasterol predominates in mesocarp [99].

Some studies have focused on the physiological phenomenon of phytosterols accumulation: biosynthesis and evolution, finding that the phytosterols amount depended on the harvest time [90,93]. Cherif et al. [90] found a relationship between the biosynthetic compounds of the glyceridic fraction of almond oil (mainly fatty acids) and those of the unsaponifiable fraction (particularly sterols). This relation may be established by 24-methylene cholesterol.

Ozcan et al. [87] compared varieties and extraction methods (cold press and Soxhlet methods). Both affected β-Sitosterol composition of the oil obtained. Neither extraction temperature nor extraction speed affected the total content of sterols in oils from the screw press, but higher temperatures caused a reduction in the content of Δ7-stigmasterol [12].

### 6.5. Sphingolipids and Phospholipids

Both components are polar lipids. Sphingolipids are complex lipids that are derived from sphingosine (unsaturated amino alcohol with 18 carbons), which is joined to a long-chain fatty acid by an amide bond forming the ceramide. Sphingolipids of plants are mainly cerebrosides (mono- and oligohexosilceramides) with a sugar molecule such as glucose, galactose, mannose and inositol. They are commonly found in cell walls, lipoproteins and other lipid-rich structures [99]. Phospholipids are a kind of lipid made up of a glycerol molecule, two fatty acids (1,2-diacylglycerol) and a phosphate group.

There are few studies about polar lipids in almond oil. Phospholipids and sphingolipids are the main classes of polar lipids with approximately 78% and 22%, respectively [100]. Between phospholipids, lecithin or phosphatidylcholine (45%), phosphatidylethanolamine or cephalin (45%), phosphatidylinositol (8%) and fosfatidiglycerol acid (2%) are the main compounds [101]. Fang [102] studied the sphingolipids content in almond kernels and found that the concentration of cerebroside (d18:2-C16:0h-glucose) was 0.068 mg/g of almond.

Compared with other nuts, almonds might not be the first choice for phospholipids, with relatively low compounds abundance and content [103]. Only 1.67% of the total fat is phospholipids, in comparison with 3.81% found in pistachios. The fatty acids of 16:0, 18:0, 18:1 and 18:2 are the most common structures of the fatty acyl moiety in almonds; phosphocholine, phosphoetanolamine and phosphoinositol are three major phospholipids species detected in almonds, representing 84% of total phospholipids.

### 6.6. Chlorophylls and Carotenoids

Tree nuts contain very low amounts of carotenoids [27]. Marginal pigments content has been found in wild almond kernels [92], and the only study that indicates the chlorophyll and carotenoid content for cultivated almond kernels is due to Ojeda-Amador et al. [35], who found 8.5–18 mg/kg of chlorophyll and 5.3–8.8 mg/kg of carotenoids in almond oils. Carotenoids concentration in almond kernels is low; consequently, it does not constitute an important dietary source of these substances [97].

### 6.7. Phenols

Phenols are the main phytochemical group and comprise the broad term “polyphenols”, which are molecules with one or more phenolic groups and one or more hydroxyl groups and comprise a large and heterogeneous group of secondary plant metabolites. They are synthesized from carbohydrates and are generally produced as defense mechanisms against pathogens and the excess of ultraviolet radiation and to attract pollinators [104]. A general description of the biosynthetic pathways and regulation of phenolic compounds in stone fruits appears in the review by Lara et al. [105]. They are responsible for the sensorial and nutritional quality and antimicrobial, antiviral and anti-inflammatory properties are also attributed to them. Beyond antioxidant properties, they also have a variety of biological activities, including antioxidant, anti-inflammatory, vasodilatory and anticarcinogenic actions and also reduce cholesterol [82,97]. In recent years, there has been an increasing interest in biological properties of natural phenolic compounds as actors in the prevention of diseases in which oxidative stress reactions are involved [106].

In an extensive review, Bolling [107] reported a total phenolic content in whole almond that ranged from 0.47 to 13.40 mg/g gallic acid equivalents (GAE); meanwhile, skinless kernels varied between 0.64 and 0.71 mg/g (GAE). Approximately 130 different polyphenols have been identified in almond, although not all of these have been quantitated. Table 5 shows the range of variability found in the scientific literature about total phenolic content, total flavonoids content and total proanthocyanidins content, and Table 6 reflects the main quantitative results about phenolic compounds in almonds. Unpeeled almond kernels have a content of total phenols higher than peeled almond kernels [97,108]. The skin represents approximately 4% of the total almond weight and contains 70–100% of total phenols present in the nut [109]. The residual cakes could be expected to possess an added value for applications in food formulations since they are a good source of phenolic compounds that concentrate in the by-product due to their polar properties [35].

The phenolic content of almond skins depends on the industrial processing used. High temperatures (i.e., blanching, drying, roasting) could promote degradation of polymeric compounds such as proanthocyanidins, hydrolysis of glycosylated flavonoids and the decomposition of aglycones, which could explain the increase observed in the content of monomeric and oligomeric flavan-3-ols after drying or roasting, and the decline in flavonol and flavanone aglycones found after these treatments [110]. The total contents of phenolic compounds identified were significantly (*P* < 0.05) higher (about 2-fold) in the roasted samples than in the blanched almonds (freeze-dried). Roasting is the most suitable type of industrial processing of almonds to obtain almond skin extracts with the greatest antioxidant capacity [111].

Cultivars, climate and geography can affect total phenols concentration in almond kernels [57,58,112,113]. For Rabadán et al. [12,70], the variability of total polyphenol content depended mainly on the crop year. The use of pesticides reduces the phenols content, so it is advisable to implement organic production [114]. Among cold-pressed oils, the press system (screw or hydraulic) and the different extraction conditions considered did not generate significative differences [11,31].

Inside the phenol groups, mainly tannins, flavonoids, phenolic acids and stilbenes can be found.

#### 6.7.1. Tannins

Proanthocyanidins are mixtures of oligomers and polymers of flavan-3-ol linked through carbon bonds, mainly C4 → C8. Tannins are divided into two groups, hydrolysable tannins and condensed tannins or proanthocyanidins (PAC). Hydrolysable tannins are derived from gallic acid and include gallotannins and ellagitannins. PACs are mixtures of oligomers and polymers of flavan-3-ol. Depending on the interflavan carbon–carbon bond, PACs could be A-type or B-type; depending on the degree of polymerization (DP), they are known as oligomeric (≤10) and polymeric proanthocyanidins (>10). In almond, most PACs are polymeric [130]. In addition, flavan-3-ol composition should be considered to determine PACs. The intrinsic complexity and diversity of almond proanthocyanidins, as well as a lack of available standards, pose analytical challenges [107].

Information about tannins is very limited and only a few studies quantify these compounds [81,107], even though PACs are the most abundant polyphenols in almond kernel, followed by flavonoids and phenolic acids. No references have been found about the presence of soluble tannins. Almond proanthocyanidins consist mainly of epicatechin and catechin, with lesser amounts of epiafzelechin. In the opinion of Bolling et al. [81,107], the cis–transconfiguration, A-/B-type ratios and flavan-3-ol types of almond PACs have not been adequately characterized.

PACs consisting exclusively of epicatechin are procyanidins (PCs). PACs containing epiafzelechin as subunits are named propelargonidins (PPs). When subunits are epigallocatechin, they are named prodelphinidins (PDs) [124].

Gu et al. [124] presented the concentrations of monomers, dimers and trimers separately because these low-molecular weight PACs oligomers (DP ≤ 3) could be absorbed intact in the gastrointestinal tract; meanwhile, PACs with DP > 3 appear not to be absorbed directly from the gastrointestinal lumen.

Table 6 summarizes the variability in tannins found in the reviewed papers published.

#### 6.7.2. Flavonoids

At least 25 different flavonoids have been identified in almonds. Anthocyanidins, flavan-3-ols, flavonols, flavanones and a biflavone have been identified in almond, almond skins, or almond blanch water [107].

Most flavonoids of almond kernel appear exclusively in the skin, while non-flavonoids phenols appear in the seed. Flavonoids of almond skin act as phytoalexins, protecting dry seeds against bacteria, fungi and other environmental stressors [132]. The flavonoids group can be divided in seven categories that include flavonols, flavones, isoflavones, flavanols, flavanones, anthocyanins and dihydrochalcones.

Flavonols were the most abundant flavonoid class in almond and include isorhamnetin-3-O-glucoside, isorhamnetin-3-O-rutinoside, kaempferol-3-O-glucoside, quercetin-3-O-glucoside, quercetin-3-O-rutinoside, galactosides and rutinosides [107]. They have been identified, but rarely quantified (Table 6).

Flavonoid composition in plants is influenced by genetic factors and environmental conditions, excluding exposure to fungi and bacteria, parasites, climate and UV light [81,112,118].

#### 6.7.3. Phenolic Acids

The presence of phenolic acids is associated with astringency, discoloration and inhibition of enzymatic activity and antioxidant properties. Phenolic acids identified in almond kernel have been caffeic, p-coumaric, ferulic, sinapic, syringic, vinyl, gallic, protocatechuic and p-hydroxybenzoic, which are fundamentally derivated from benzoic acid or cinnamic acid.

Senter and Horvat [131] found that protocatechuic acid is the dominant phenolic acid in the edible part of almond kernel, followed by p-hydroxybenzoic acid and vinyl acid. Wijeratne et al. [108] observed that the total quantity of free phenolic acids was 163 mg/100 g in the skin, while total quantities of esterified phenolic acids obtained from skin, shell and whole seed were 2796, 1671 and 400 mg/100 g, respectively. Monagas et al. [111] compared the polyphenol concentration in the almond skin of Spanish and American almonds (Table 6).

Bolling [107] reported the almond skin content of protocatechuic acids. The hydroxycinnamic acids, chlorogenic acid, ferulic acid, caffeic acid, sinapic acid and two diferulates have been identified in almond skin, although only chlorogenic acid, caffeic acid and p-coumaric acid have been quantitated.

#### 6.7.4. Stilbene

Among stilbenes, resveratrol dominates, acting as a phytoalexin. Recently, Xie and Bolling [25] characterized stilbenes in Californian almond cultivars, concretely resveratrol-3-β-glucoside, in concentrations of 7.19 to 8.52 mg/100 g almonds. Similar to other polyphenols, stilbenes were concentrated in skins.

## 7. Volatile Compounds

Seventeen aroma compounds were detected in raw almonds [133], including six aldehydes, two ketones, two nitrogen-containing compounds, one sulfur-containing compound, two acids, one furanone and three unknown compounds. Six of these compounds were quantitated in raw almonds, where vanillin with 830 ng/g was the most abundant and acetic acid (137 ng/g) and nonanal (72 ng/g) were found in high abundance.

Ojeda-Amador et al. [35] analyzed volatile compounds which are related to sensory notes, such as fruit/banana (hexanol), oily/green–sweet (hexanal), fruity (pentanol) and bitter almonds (benzaldehyde). The most important family found in all the varieties studied was that of aldehydes (1.35–7.52 mg/kg). Benzaldehyde was the main aldehyde (52–74% of total), followed by hexanal (0–10%).

Alcohols were the second major family, accounting for 14% to 30% of the total volatiles. Hexanol was the main contributor and was most abundant in “Marcona” (1.89 mg/kg). Acids (mainly acetic acid), hydrocarbons, ketones and terpenes showed close concentrations to each other, indicating about 0.30 mg/kg for each family.

## 8. Conclusions

Almond kernel contains a considerable amount of good-quality proteins, mainly globulins, essential minerals and fiber with a low content in sugars, in addition to many phytochemicals with potential health benefits. The presence of large variability in nutritive compounds has been reported, although most pre- and postharvest factors may have a significant effect on their content. However deeper studies about drying, blanching, storage or roasting processes and genetic, agricultural and environmental conditions are necessary to clarify their influence on the quality and quantity of almond phytochemicals.

As regards the bibliography consulted, practically no work has been found focused on the study of the phenotypic correlations that occur between the different components of the almond.

The complexity in the phytochemical composition makes the use of standard methods for extracting and quantifying almond phytochemicals difficult. Non-conventional extraction techniques are gaining major interest, especially methods based on microwave, supercritical fluids and ultrasound, combined with well-known and safe solvents such as ethanol, water and ethanol−water mixtures. Other methods such as sonication and hydrolysis are barely cited in scientific papers.

In another direction, more studies are needed to understand the impact of almond processing on protein and AA digestibility. Furthermore, increasing efforts to establish a new method for assessing protein quality, based on the Digestible Indispensable Amino Acid Score (DIAAS) system, are necessary.

The valorization of non-lipid compounds from almond has been scarcely treated in the scientific literature. Most papers focus on compounds identification and quantification and rarely on industrial extraction methods, as opposed to oil extraction. However, the nutritional composition of the non-lipid fraction of almond kernel makes the by-products obtained in the oil extraction process interesting candidates for food applications, to be used as a source of protein, fiber and minerals.

## Figures and Tables

**Table 1:** Macronutrients content (%) of almond samples with different origins.

Nutrient	Range of Variability (g/100 g)	Origin	Source of Variability Studied			References
			V *	E **	Ap ***	
Protein, total (N × 5.18)						
	16.4–22.1	USA	-	-	-	[38]
	18.5–24.0	California	Yes	Yes	-	[9]
	20.7–23.3	USA	Yes	-	-	[45]
	15.8–25.1	Spain	Yes	Yes	-	[18]
	14.5–29.2	Spain	Yes	Yes	-	[39]
	8.4–24.7	Spain	Yes	-	-	[14]
	14.1–26.5	Spain	-	-	-	[3]
	21.0–24.0	Portugal	Yes	Yes	-	[46]
	9.6–28.5	France, Italy and Greece	Yes	Yes	-	[41]
	20.0–32.8	Spain and Morocco	Yes	Yes	-	[39]
	14.1–35.1	Morocco	Yes	Yes	-	[42]
	16.7–31.5	Turkey	Yes	Yes	-	[47]
	12.7–16.3	Turkey	Yes	-	-	[40]
	20.4–25.8	Turkey	Yes	Yes	-	[48]
	11.52 ± 1.1	Nigeria	-	-	-	[49]
	23.8	India	-	-	-	[50]
	20.0	South Africa	-	-	-	[51]
	17.36–23.02	Serbia	Yes	-	Yes	[52]
Carbohydrates, total						
	14–21	Portugal	Yes	Yes	-	[46]
	23.6–27	USA	Yes	-	-	[45]
	28	Nigeria	-	-	-	[53]
	28.0	South Africa	-	-	-	[51]
Sugars, soluble						
	2.6	Turkey	Yes	-	-	[54]
	7.9	Spain	Yes	-	-	[15]
	1.74–4.31	Greece	Yes	-	Yes	[55]
Sucrose						
	2.5–5.1	California	Yes	Yes	-	[9]
	1.42–3.62	Greece	Yes	-	Yes	[55]
	1.15–2.22	Portugal	Yes	-	-	[56]
	3.67–7.09	Spain	-	-	Yes	[57]
	1.21–3.08	Portugal	Yes	-	-	[58]
Starch						
	0.4–1.4	Italy	-	-	-	[59]
Fiber, total dietary						
	9.8	California	-	-	-	[60]
	7.9–16	California	Yes	Yes	-	[9]
	3.3–8.6	Spain	Yes	Yes	-	[22]
	4.73–6.01	Spain	-	-	Yes	[57]
	11–14	Italy	-	-	-	[59]

* Variety; ** Environment/crop year; *** Agronomic practices (irrigation, fertilization, etc.).

**Table 2:** Average value or range of main mineral elements (macro- and microminerals) found in almond kernel in the literature (mg/100 g).

Ash (g/100 g)	K	P	Ca	Mg	S	Cl	Na	Fe	Cu	Mn	Zn	Origin	Reference
	435	577	298	299	587		2.27	3.4	0.96	1.36	3.04	Spain	[7]
2.69–3.6	821	585	275	281	130	14	10.8	4	1.2	1.6	3.8	Spain	[15]
	618–785	345–507	88–124	242–285			4.7–15.5	3.5–5.3	1–1.6	1.1–1.7	3.4–3.9	Spain	[18]
2.74–3.05	1373.8	873.8	243.2	351			32.6	23.4	1		5	Turkey	[40]
	1546–1685	253–259	640–678	447–494				5.5–6.5	2.4–2.6	3.8	7.6–8.0		[63]
	1050	300	467	30				7.0	0.5		3.4	Italy	[74]
3.03–4.66	1677–2051	404–800	98–187	361–513			5.66–10.38	3.98–14.6	1.60–2.30	2.90–3.39	7.78–8.84	Turkey	[54]
-	465–1235	119–748	160 -663	100–333								France, Italy and Greece	[41]
2.3–3.4	543–902	364–548	198–373	224–303				2.58–4.47	0.46–1.57	1.31–3.98	2.02–4.03	California	[9]
3.29–4.66	679–986	584–697	250–332	325–381			9.20–16.06	6.08–10.62	2.02–3.97	2.52–4.76	4.80–9.53	Turkey	[48] *
5.0			539.2	542.4				7.15	2.37	2.58	4.97	South Africa	[51]
		133.25	450.0					6.25				India	[76]

* Referred to non-dried matter. K: potassium; P: phosphorus; Ca: calcium; Mg: magnesium; S: sulfur; Cl: chloride; Na: sodium; Fe: iron; Cu: copper; Mn: manganese.

**Table 3:** Vitamin content in almonds.

Nutrient	Range of Means		Origin	Variability Sources			References
	mg/100 g Almonds	mg/100 g Almond Oil		Variety	Environment	Extraction Method	
Vitamin E homolog							
Total tocopherols		50.1–49.0	Spain	Yes	Yes	-	[12]
α-tocopherol		24.2	-	-	-	-	[84]
		17.4	-	-	-	-	[85]
		37.0–57.1	Argentina	Yes	Yes	-	[30]
		18.0–32.0	California	Yes	Yes	-	[9]
		30.9–65.7	Morocco	Yes	Yes	-	[42]
		31.3–54.6	Morocco	Yes	Yes	-	[86]
	34.9		California	-	-	-	[83]
	5.96–19.42		Portugal	Yes	-	-	[58]
		27–38	Portugal	Yes	Yes	-	[46]
		42.0–54.2	Spain	Yes	-	-	[35]
	23.7–37.4		Spain	Yes	-	-	[35]
		14.18–17.96		Yes	-	Yes	[87]
β-tocopherol		3.1	-	-	-	-	[84]
		1.7	-	-	-	-	[85]
		0.18–0.24	Portugal	Yes	Yes	-	[46]
ϓ-tocopherol		3.1	-	-	-	-	[84]
		5.7	-	-	-	-	[85]
		0.54–4.25	Morocco	Yes	Yes	-	[86]
		0.7–2.1	Portugal	Yes	Yes	-	[46]
	1.4		California	-	-	-	[83]
		0.67–2.79	Spain	Yes	-	-	[35]
	0.17–1.4		Spain	Yes	-	-	[35]
δ-tocopherol		n.d.	-	-	-	-	[84]
		1.7	-	-	-	-	[85]
		0.017–0.24	Morocco	Yes	Yes	-	[86]
		0.02–0.05	Portugal	Yes	Yes	-	[46]
α-tocotrienol		Traces	-	-	-	-	[85]
		0.04–0.2	Portugal	Yes	Yes	-	[46]
		0.3–0.5	Spain	Yes	-	-	[35]
ϓ-tocotrienol		0.11–0.24	Portugal	Yes	Yes	-	[46]
Other vitamins							
Biotin	0.01–0.05		California	-	-	-	[17]
	0.12–0.90		Italy	-	-	-	[88]
Folate	0.10–0.13		California	-	-	-	[17]
Niacin (B3)	3.3–3.7		California	-	-	-	[17]
	1.5–3.4		Italy	-	-	-	[88]
		1.40–5.02	California	Yes	Yes	-	[9]
Pantotenic acid	0.36–0.38		California	-	-	-	[17]
Pyridoxine (B6)	0.16		California	-	-	-	[17]
	0.186		California	-	-	-	[83]
Riboflavin (B2)	1–1.1		California	-	-	-	[17]
		0.58–2.27	California	Yes	Yes	-	[9]
	1.432		California	-	-	-	[83]
Thiamine (B1)	0.19–0.25		California	-	-	-	[17]
	0.192		California	-	-	-	[83]

**Table 4:** Minor compounds: phytosterols, terpenic alcohols, squalene, aliphatic alcohols and tocopherols.

Compound	µg/g (%)	References
**Desmethylsterols**		
Cholesterol	n.d.–7.18 (0.25)	[85,92,93,94]
24-Methylene-cholesterol	1.15 (0.04)–3.9	[93,94]
Campesterol	49–134 (2.46–16.7)	[11,12,85,92,93,94,95]
Campestanol	3.73 (0.13)-33	[94,96]
Stigmasterol	3.9–50 (0.41–6,9)	[85,92,93,94,95,96]
Δ7-Campesterol	22.39 (0.78)	[94]
Δ5,23-Stigmastadienol + Clerosterol	30.8–40.19 (1.40)	[93,94]
β-Sitosterol	580–2290 (72.4–95.5)	[11,12,85,87,92,93,94,95,96]
Sitostanol	32–54.83 (1.91)	[93,94,96]
Δ5-Avenasterol	32–283.89 (3.52–9.89)	[85,92,93,94,95,96]
Δ5,24-Stigmastadienol	6.05–42.48 (0.29–1.48)	[92,93,94]
Δ7-Stigamasterol	9.74–55.69 (0.43–1.94)	[12,93,94]
Δ7-Avenasterol	5.42–39.90 (0.26–1.39)	[11,93,94]
Total µg/g	1222–2870	[11,12,85,92,93,94,96,97]
**Methylsterols**		
Obtusifoliol	7.90 (26.57)	[94]
Gramisterol	4.00 (13.46)	[94]
Citrostadienol	17.83 (59.96)	[94]
**Dimethylsterols**		
Dammaradienol	0.62 (7.44)	[94]
Taraxerol	0.38 (4.55)	[94]
α + β Amyrin	2.08 (24.97)	[94]
Cycloartenol	0.99 (11.91)	[94]
24-Methylencycloartanol	4.27 (51.13)	[94]
Total sterols	2908.56	[94]
Squalene µg/g	37.9–114.2	[30,90,94,98]
**Terpenic alcohols**		
Phytol	(71.65)	[94]
Geranylgeraniol	(28.35)	[94]
Total µg/g	9.74	[94]
**Aliphatic alcohols**		
C22-OH	(23.13)	[94]
C23-OH	(2.56)	[94]
C24-OH	(29.66)	[94]
C25-OH	(7.70)	[94]
C26-OH	(40.31)	[94]
Total µg/g	5.55	[94]

In parenthesis: samples origin country/region; in brackets: source of variability studied. [a] variety; [b] environment/crop year; [c] extraction method. [94]: (Brazil); [92]: (Iran); [85]: (Sweden); [30]: (Argentina) [a,b]; [93]: (Turkey) [c]; [95]: [c]; [87]: (Turkey) [c]; [96]: (USA); [11]: (Spain) [a,b]; [12]: (Spain) [c]; [90]: (Tunisia) [a,b].

**Table 5:** Total phenolic content (mg/g) gallic acid equivalents (GAE), total proanthocyanidins (mg/100 g) and total flavonoids (mg/100 g) in almonds, almond oil and defatted almond cake.

Range of Means				Origin	Variability Sources			References
Almonds	Almond Skins	Almond Oil	Defatted Almond Cake		Variety	Environment	Extraction Method	
Total phenolic content (mg/g) gallic acid equivalents (GAE)								
4.18				USA	-	-	-	[115]
1.27–2.41	0.099–0.268			California	Yes	-	-	[109]
1.30–4.56				Austria	Yes	-	-	[84]
0.45–0.49 ^a^				Austria	Yes	-	-	[84]
1.10–2.90								[97]
0.09–1.63				Portugal	Yes	Yes	-	[112]
	27.1–59.1			Morocco	Yes	-	-	[113]
		0.019–0.022 ^b^		Spain			Yes	[31]
		0.0085–0.0324		Spain	Yes	Yes	Yes	[11]
		0.019–0.026		Spain	Yes		Yes	[12]
0.03–0.81				Portugal	Yes	-	-	[58]
0.71–1.26		0.003–0.006	0.82–2.06	Spain	Yes	-	Yes	[35]
0.20–1.39				Serbia	Yes	-	-	[116]
Total proanthocyanidins (mg/g)								
	0.15–48.80			Spain	-	-	Yes	[117]
	5.81–28.80			Spain	-	-	Yes	[118]
0.70–2.90 ^c^				-	-	-	-	[62]
	5.00–25.00			-	-	-	Yes	[119]
Total flavonoids (mg/g)								
6.24–25.02				Portugal	Yes	-	-	[112]
	84.68–237.20 ^d^			Portugal	Yes	-	-	[56]
	14.1–25.7			Morocco	Yes	-	-	[113]
n.d.–5.45								[120]
12.88–19.49				Portugal	Yes	-	-	[58]

a: blanched kernels without skin; b: caffeic acid equivalents; c: total tannins; d: hull extract; n.d.: not detected.

**Table 6:** Phenolic compounds quantified in almonds and almond skins (mg/100 g).

Compound		Almond	skin	References
Flavonoids				
Flavan-3-ol	(+)-Catechin	0.1–36.6	0.69–18.4	[52,109,110,111,116,118,120,121,122,123,124,125]
	(-)catechin gallate	0.68–1.04		[52,126,127]
	Dihydrokaempferol	0.04–9.8	4.99–6.02	[111,118,126]
	Dihydroquercetin	0.51–1.60	n.d.–1.61	[110,111,118,128]
	(-)-Epicatechin	0.03–26.6	0.13–11.0	[52,58,109,110,111,118,120,121,122,123,128,129]
	Epicatechin gallate	1.34–2.60		[52,126,127]
	Gallocatechin gallate	n.d.–0.104		[58]
	(-)epigallocatechin gallate	1.04–1.60		[52]
	(-)epigallocatechin	8.07–8.87		[52]
	(-)gallocatechin	1.17–3.26		[52]
Flavanone	Eriodictyol	n.d.–0.46	n.d.–0.78	[58,110,111,118,120,126,128]
	Eridictyol-7-O-glucoside	n.d.–0.49	0.04–3.38	[58,110,111,118,120]
	Naringenin	0.01–9.74	0.03–20.6	[58,110,111,116,118,120,126,127,129]
	Naringenin-7-O-glucoside	0–5.88	0.04–14.3	[58,110,111,118,120,126,127,129]
Flavonol	Isorhamnetin	0.005–3.20	0.40–4.55	[58,110,111,118,120,124,125,126,127,128,129,130]
	Isorhamnetin-3-O-galactoside	0.30–0.92		[111,118]
	Isorhamnetin-3-O-glucoside	n.d.–14.9	0.20–16.9	[58,110,120,126,127,129]
	Isorhamnetin-3-O-rutinoside	n.d.–74.1	0.53–75.7	[58,110,111,118,120,126,127,128,129]
	Kaempferol	n.d.–0.49	0.01–1.25	[110,111,116,118,120,123,124,125,126,128,129,130]
	Kaempferol-3-O-galactoside	n.d.–2.17	0.72–1.15	[111,118,126]
	Kaempferol-3-O-glucoside	n.d.–3.77	n.d.-39.0	[58,110,111,118,120,126,128,129]
	Kaempferol-3-O-rutinoside	n.d.–23.3	0.10–23.9	[58,109,110,111,118,120,126,127,128,129]
	Quercetin	n.d.–3.58	0.03–0.70	[110,111,118,120,123,124,125,126,128,129,130]
	Quercetin-3-O-galactoside	0.24–1.37	n.d.-1.34	[109,110,111,118,120,126]
	Quercetin-3-O-glucoside	0.04–0.16	n.d.-0.90	[109,110,111,118,120,129]
	Quercetin-3-O-rutinoside	n.d.–1.66	n.d.-41.2	[58,111,118,120,126,127,129]
Phenolic acids/aldehydes				
Hydroxybenzoic acid	p-Hydroxybenzoic acid	n.d.–1.23	0.03–1.90	[58,110,111,116,118,120,126,129,131]
	Gallic acid	0.05–1.61	n.d.–1.61	[58,123,131]
	Protocatechuic acid	n.d.–6.19	0.04–4.46	[58,110,111,116,118,120,131]
	Vanillic acid	n.d.–0.30	0.01–5.81	[58,110,111,116,118,120,124,125,130,131]
	Ellagic acid	n.d.–0.135		[116]
Hydroxybenzoic aldehyde	Protocatechuic aldehyde	2.52–5.77	0.25–2.17	[110,111,118]
Hydroxycinnamic acid	Chlorogenic acid	n.d.-2.29	0.17–9.57	[58,110,111,116,118,120,123]
	Caffeic acid	0.11–3.21	n.d.–3.21	[116,123,125]
	o-Coumaric acid	0.22–0.69		[120,123,124,125,130]
	p-Coumaric acid	0.01–0.59	n.d.–0.37	[58,110,111,116,118]
	Ferulic acid	0.02–2.15		[116,125]
Proanthocyanidins				
	A-type trimers		0.16–0.53	[111,118]
	Procyanidin B1	1.69–7.28		[111,118,121],
	Procyanidin B2	0.03–8.30	0.23–3.39	[110,111,118,122]
	Procyanidin B3	0.19–0.45		[111,118,121,124,127]
	Procyanidin B3+B1		0.30–2.96	[110,111,118,121,122,124,125]
	Procyanidin B5	n.d.-0.43	0.23–1.51	[110,111,118,121,122,124]
	Procyanidin B7	0.28–1.43	0.37–2.47	[110,111,118,121,122,124]
	Procyanidin C1		0.11–2.55	[110,121,122,124]
Tannins				
	PAC dimers	4.00–18.7		[110,122,124]
	PAC trimers	2.70–14.0		[122,124]
	PAC 4–6 mers	7.00–51.4		[122,124]
	PAC 7–10 mers	9.60–52.0		[122,124]
	PAC polymers	43.9–121		[122,124]
